# Systematic review of trends in deep learning for UAV cybersecurity

**DOI:** 10.3389/frai.2026.1752124

**Published:** 2026-05-15

**Authors:** Usman Tariq, Tariq Ahamed Ahanger, Irfan Ahmed

**Affiliations:** 1 Prince Sattam Bin Abdulaziz University, Al-Kharj, Saudi Arabia; 2 Department of Computer Science, College of Engineering, Virginia Commonwealth University, Richmond, VA, United States

**Keywords:** adversarial robustness, deep learning, explainable AI (XAI), intrusion detection systems, UAV cybersecurity

## Abstract

Unmanned Aerial Vehicles (UAVs) operate in navigation, sensing, and communication environments that are frequently degraded or adversarial. Their attack surface spans flight-control and payload software, radio links, and swarm coordination. This PRISMA-aligned systematic review synthesizes peer-reviewed studies published between 2015 and 2025 and organizes the evidence using an OSI-inspired threat taxonomy that maps spoofing, jamming, intrusion, and malware to system touchpoints and observable anomalies. We compare deep learning architectures, training targets, feature representations, evaluation practice, and deployment constraints relevant to single UAVs and swarms. Across the literature, convolutional and recurrent models dominate intrusion and anomaly detection pipelines, while attention-based, graph, and generative models appear in newer work targeting multi-agent settings and limited labels. Evidence most often relies on protocol traffic and onboard telemetry, whereas RF inputs are used less frequently and are typically represented as raw samples or spectrograms when datasets allow. Studies increasingly report efficiency-oriented deployment using pruning, quantization, distillation, or split inference to meet onboard compute and energy limits. Federated and multi-agent approaches are evaluated for scalability and robustness under poisoned updates, and blockchain-integrated designs are discussed under bandwidth and power constraints. Key gaps persist in shared datasets, repeatable adversarial stress testing, uncertainty and explainability reporting, privacy preservation, and certification-ready assurance cases for aviation regulation.

## Introduction

Unmanned Aerial Vehicles are increasingly embedded in critical infrastructure sectors and coordinated networks, often termed the Internet of Drones (IoD). This rapid expansion of drones in domains like defense, public safety, logistics, and agriculture has markedly increased their exposure to cyber threats (Rugo and Ioini, 2022). By connecting UAVs via wireless links and cloud systems, IoD architectures broaden the attack surface. This means a drone’s vulnerability extends beyond the aircraft itself to encompass communication networks and ground control stations (Wang et al., 2023). Consequently, attackers can target various entry points, such as navigation signals, control links, firmware, or APIs, to hijack or disrupt UAV operations. Modern UAV cyber threats range from signal interference (GPS/BeiDou spoofing feeding counterfeit coordinates, and radio-frequency jamming of control channels) to network intrusions, denial-of-service attacks, and malware infiltration of on-board systems (Medhi et al., 2025). After in-depth investigation, we illustrate Table 1 to present a layered taxonomy of vulnerabilities and anomalies affecting both individual UAVs and UAV swarms, and thus systematically organize it according to the seven layers of the OSI (Open Systems Interconnection) model to illustrate their respective attack vectors and observable deviations. We noticed that adversarial induced exploits could lead to mission failures, loss of sensitive data, or even physical hazards. Indeed, by exploiting inbuilt vulnerabilities, the adversaries can easily jam a UAV’s satellite link and spoof its GNSS (Global Navigation Satellite System) guidance to silently reroute a surveillance drone into hostile hands. These risks underscore the urgent need to fortify UAV ecosystems against an evolving spectrum of cyberattacks.

**Table 1:** Taxonomy of UAV and UAV swarm vulnerabilities and anomalies (by OSI Layer).

OSI layer	Single UAV: vulnerabilities	Single UAV: anomalies	UAV swarm: vulnerabilities	UAV swarm: anomalies
Physical	GPS Spoofing, GNSS Jamming, Electromagnetic Interference (EMI), Acoustic Sensor Hijack, Signal Replay, Power Supply Manipulation	Loss of GPS Lock, Altitude Drift, Heading Instability, Erratic Motor RPM (revolutions per minute), Position Desync	Coordinated GNSS Deception, Distributed Jamming, Power Signal Saturation, RF Flooding	Swarm Drift, Desynchronized Flight Paths, Collective Loss of Navigation, Power Overload Alerts
Data Link	De-authentication Attack, MAC (media access control) Spoofing, Channel Saturation, Frame Injection, Replay Attack	Packet Drop Burst, Link Latency Spikes, Unexpected Retransmissions, Sudden Link Loss	Sybil Attack, MAC Cloning, Flooding Control Channel, Compromised Link Allocation	Link Reconfiguration Loops, Node Isolation, Inter-UAV Link Loss, Channel Instability
Network	IP Spoofing, Routing Manipulation, DoS (Denial-of-Service)/DDoS (Distributed Denial-of-Service), ARP (address resolution protocol) Poisoning, Packet Injection	Routing Loop, Unreachable Host Errors, Unexpected IP Reassignment, Network Congestion	Blackhole Attack, Wormhole Attack, Sinkhole Attack, Cooperative Routing Manipulation	Routing Oscillation, Packet Storms, Swarm Routing Divergence, Latency Clusters
Transport	TCP SYN Flood, UDP Flooding, Port Scanning, Session Hijacking	Port Unavailability, Connection Timeout, Retransmission Burst, Buffer Overflow	Coordinated SYN Flood, Multi-node UDP Amplification, Distributed Session Hijack	Collective Communication Delay, Cascading Timeout Events, Cross-Node Retransmission Storm
Session	Command Injection, Session Replay, Token Theft, Session Reset	Unauthorized Session Start, Unrecognized Command Patterns, Session Timeout Spike	Session Key Compromise, Distributed Token Forgery, Cross-Session Injection	Desynchronized Session Keys, Multiple Unverified Sessions, Command Authentication Failure
Presentation	Data Format Corruption, Protocol Exploit, Payload Encoding Attack	Decoding Failure, File Parsing Error, Data Serialization Fault	Model Poisoning, Cooperative Payload Injection, Encrypted Payload Tampering	Corrupted Shared Models, Inconsistent Data Encoding, Collective Parsing Errors
Application	Malware Injection, Firmware Tampering, API Exploit, Backdoor Activation, Insider Attack	Unresponsive Control Interface, Unexpected Flight Commands, Unauthorized API Calls	False Data Injection, Consensus Manipulation, AI (artificial intelligence) Model Poisoning, Byzantine Node	Divergent Mission Goals, Inconsistent Threat Decisions, Swarm Split Behavior, False Consensus Detection

To counteract these vulnerabilities, researchers are increasingly turning to artificial intelligence, particularly deep learning as a linchpin of UAV cybersecurity defenses (Zeng and Nait-Abdesselam, 2025). Deep learning techniques offer the ability to automatically learn complex patterns of normal versus malicious UAV behavior to support intrusion detection systems to flag anomalies in real time that would elude static rule-based methods (Achuthan et al., 2024). A wide range of neural network architectures has now been explored for UAV security, including convolutional and recurrent networks for analyzing telemetry streams, autoencoders for anomaly detection, generative models for attack simulation, and even deep reinforcement learning for autonomous threat response (Islam et al., 2025). These data-driven models have demonstrated substantial gains in detection capability, successfully identifying spoofing, jamming, and hijacking attempts with higher accuracy and speed than conventional signature-based approaches. In particular, deep neural detectors can recognize subtle deviations in UAV control traffic indicative of an attack and issue alerts or autonomously initiate protective maneuvers. By leveraging onboard processing and edge AI, such systems can respond to threats within the UAV or swarm itself without always relying on human operators or constant cloud connectivity (Alsadie, 2025). As a result, deep learning is now perceived as a primary defense to enhance the resilience of drone networks against sophisticated cyber incursions. Nonetheless, despite this progress, many open questions remain regarding how to best design, optimize, and govern AI-driven UAV security solutions.

Accordingly, this systematic review is organized around five core research questions (RQ-1 to RQ-5) that address these gaps. Together, they examine the evolution of deep learning architectures for UAV threat detection, the deployment of efficient models on resource-constrained drones, the integration of learning-based defenses with distributed frameworks, the robustness of models against adaptive attackers, and the broader ethical/regulatory context for AI in UAV security.

*RQ1*: How have deep learning architectures evolved to counter the diverse spectrum of UAV cybersecurity threats, including intrusion, spoofing, jamming, and malware, while achieving measurable improvements in detection accuracy, latency reduction, and model compactness over the past decade?

*RQ2*: What optimization and deployment strategies enable lightweight, energy-aware, and real-time deep learning models to operate effectively on resource-constrained UAVs and swarm platforms without compromising detection reliability or communication integrity?

*RQ3*: In what ways can hybrid and decentralized security frameworks integrating deep learning with federated, edge, fog, or blockchain infrastructures enhance data integrity, cross-UAV trust management, and resilience against coordinated adversarial attacks?

*RQ4*: How can adaptive and self-defending deep learning paradigms (e.g., adversarially trained, transfer-learned, or self-supervised models) improve robustness, generalization, and rapid response to emerging and zero-day cyber threats within dynamic UAV environments?

*RQ5*: What ethical, regulatory, and explainability frameworks are necessary to ensure transparent, accountable, and privacy-preserving deployment of AI-driven UAV defense systems in compliance with aviation cybersecurity standards and societal trust requirements?

As evident from RQ-1 to RQ-5, relative to existing UAV cybersecurity surveys, the unique contribution here is that the synthesis is anchored in deep learning evidence and explicitly ties each claimed gain to its research setting (data source, evaluation protocol, and runtime constraints), which helps distinguish transferable deployment guidance from results that are only valid under a specific pipeline.

## Background and preliminaries

**Figure 1:**
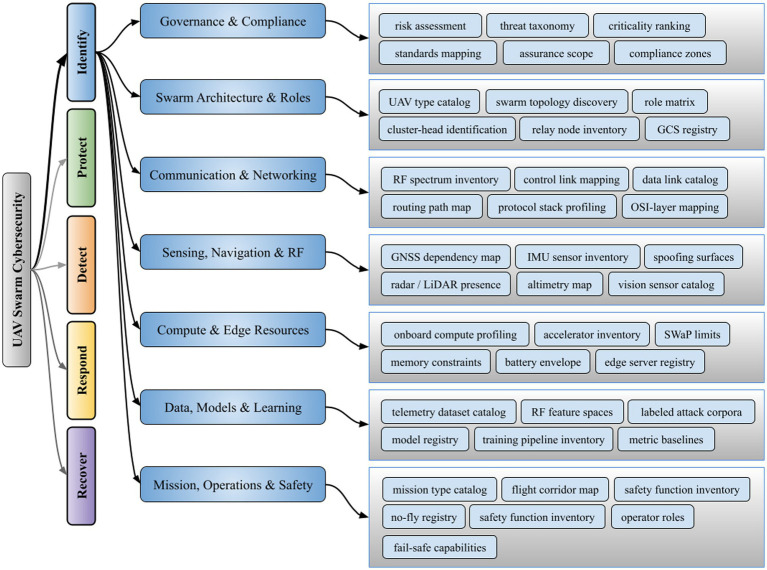
Taxonomy of identify functions for UAV swarm cybersecurity.

In a UAV swarm, multiple drones collaborate in formations or as *ad hoc* networks, even serving as aerial base stations to extend communications coverage (Wang et al., 2024). While these architectures promise enhanced capability and scalability, connecting drones into IoD ecosystems also broadens their attack surface beyond the individual aircraft to encompass inter-UAV links, ground control stations, and supporting cloud infrastructure (Bai et al., 2024). As a result, modern UAV networks face an expanded vulnerability footprint (i.e., as illustrated in Figure 1) that demands rigorous cybersecurity measures from the outset.

The UAV cybersecurity threat landscape spans every component of a drone system. As indicative by Table 1, the vulnerabilities arise across hardware, software, communication, and sensor subsystems and thus each offer potential avenues for exploitation (Mynuddin et al., 2024). For instance:

- Communication link attacks are a prominent concern, in which the wireless control channels can be jammed to disrupt operator commands or flooded with rogue traffic to degrade network performance.
- Adversaries may/can also mount eavesdropping and man-in-the-middle attacks on UAV data links by intercepting telemetry or injecting spoofed commands if communications lack strong encryption and authentication.
- Meanwhile, sensor and navigation threats exploit the drone’s reliance on positioning and onboard sensors. GPS/GNSS signals can be spoofed or blocked by feeding the UAV counterfeit coordinates or denying location fixes. Such attacks induce errant navigation or loss of control, which potentially can divert a drone into no-fly zones or hostile capture.
- At the software and control level, attackers target the UAV’s firmware and mission software with malware or code injection. By exploiting unpatched vulnerabilities or insecure interfaces, an adversary can hijack the flight controller, disable safety features, or exfiltrate sensor data.

Indeed, documented incidents and experiments show that signals and commands can be maliciously manipulated to cause mission failures or unsafe behaviors in both single drones and coordinated swarms (Bai et al., 2024). These multifaceted threats highlight the urgent need to harden UAV architectures against attacks at all layers of operation.

In response to broad attack surface, as indicated in the preceding section, the researchers are increasingly turning to deep learning as a cornerstone of UAV cybersecurity defenses (Medhi et al., 2025; Mynuddin et al., 2024). Unlike static rule-based systems, machine and deep learning methods can automatically learn the complex patterns that distinguish normal UAV behavior from malicious activities, and it supports more adaptive and timely threat detection (Al-Syouf et al., 2024; Morshedi and Matinkhah, 2025). A wide range of neural network architectures have been applied to UAV security tasks. CNN and RNN have been trained on UAV telemetry and network traffic to identify telltale signs of intrusions or anomalies in real time (Gamal et al., 2024; Ahmad et al., 2025). Autoencoders and other unsupervised deep models have been used for anomaly detection by learning a drone’s normal sensor data patterns and flagging deviations that may indicate spoofing or tampering (Kirubhakaran et al., 2025). Researchers have also explored generative models to simulate attack scenarios for training, and deep reinforcement learning (DRL) to develop UAVs that autonomously adjust their behavior under attack (e.g., evasive maneuvers under jamming or dynamic re-routing when certain nodes are compromised). Our in-depth investigation revealed that these data-driven approaches have demonstrated significant gains in detection accuracy and speed and often outperformed traditional signature-based or statistical methods in identifying GPS spoofing, signal jamming, and command injection attempts (Al-Haija and Badawi, 2022).

We have also observed that a key enabler for these deep learning defenses is the ability to deploy models on resource-constrained UAV platforms and edge nodes. Given the strict payload, power, and latency limitations of drones, substantial research effort has focused on edge inference optimizations for onboard models (Hussain et al., 2024). Techniques such as model pruning and knowledge distillation have been used to compress deep neural networks while preserving their threat detection performance (Wisanwanichthan and Thammawichai, 2025). By reducing model size and computation, UAVs can run intrusion detection or anomaly recognition algorithms locally in real time without constant reliance on cloud support. This empowers drones and swarms to detect and respond to threats autonomously during missions.

Indeed, modern UAV security frameworks leverage onboard threat mitigation triggered by AI-driven detectors: when a deep learning model flags a malicious event (e.g., a sensor spoof or a network intrusion), the UAV can immediately initiate protective actions (Alsadie, 2025; Tlili et al., 2024; Yang et al., 2025). Such actions may include isolating a compromised subsystem, switching to backup control links, executing an emergency landing or return-to-base maneuver to prevent further damage (Jasim et al., 2021).

By coupling detection with automated response, deep learning-based solutions aim to contain attacks *in situ* to enhance the resilience of both individual drones and large-scale drone swarms. As a result, artificial intelligence and deep learning in particular is viewed as indispensable for securing next-generation UAV networks (Liu et al., 2020; Sarkar and Gul, 2023). Though, these advancements create new challenges in model robustness, real-time performance, and trustworthiness that drive research toward optimized and verifiably safe AI-based UAV cybersecurity methods. Some of challenges include but are not limited to: adversarial vulnerability of deep models, limited computational resources on UAV hardware, latency constraints in real-time inference, scarcity of labeled attack datasets, difficulty in generalizing across diverse UAV platforms, communication bottlenecks in distributed learning, explainability and transparency limitations, susceptibility to model poisoning or backdoor attacks, and lack of standardized evaluation benchmarks.

## Systematic review methodology

**Figure 2:**
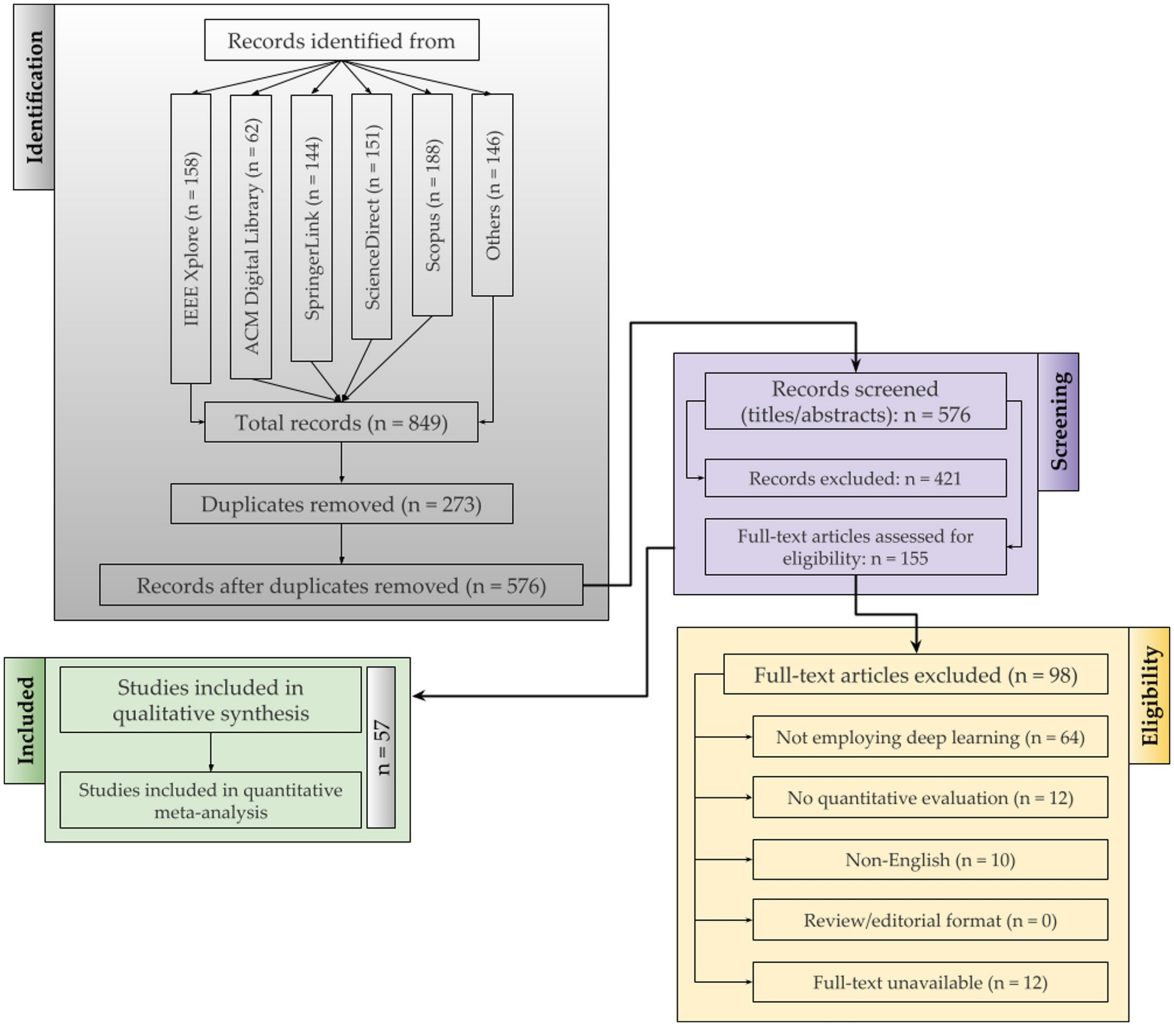
Identification, screening, and inclusion of studies using the PRISMA framework.

This review follows a structured and transparent protocol to identify, screen, and synthesize peer-reviewed research studies related to deep learning applications for UAV cybersecurity. The methodology aligns with PRISMA-based systematic review principles (Shamseer et al., n.d.) and focuses on quantitative and qualitative evidence across five predefined research questions (i.e., RQ-1 to RQ-5). It is important to note that this review was not registered in a formal protocol registry such as PROSPERO. This decision reflects the scope limitations of PROSPERO, which is designed for systematic reviews in health-related, social care, and public health domains, and does not accommodate technical or engineering-focused evidence syntheses. As the present research framework integrates computer science and electrical engineering perspectives on deep learning architectures for UAV cybersecurity, it falls outside the eligibility criteria of PROSPERO, and to the best of our knowledge, no equivalent prospective registry currently exists for this domain. Yet, methodological rigor was maintained through a clearly structured review design. The research questions, inclusion criteria, and screening procedures were defined in advance. Hereby, we have strictly followed following principles:

- The inclusion criteria (i.e., as indicated in Table 2) restricted the selection to mostly peer reviewed journal articles indexed primarily in ACM, IEEE Xplore, SpringerLink, Elsevier, and Wiley published predominantly between 2015 and 2025.
- Studies based on simulations, real-world UAV datasets, or hybrid swarm environments were prioritized. Only works with explicit architectures, performance metrics, or comparative analyses were included.
- Patents were excluded.
- The search strategy employed Boolean operators integrating keywords such as ‘UAV’, ‘cybersecurity’, ‘deep learning’, ‘intrusion detection’, ‘spoofing’, ‘federated learning’, and ‘adversarial robustness’. The complete database-specific Boolean search strings, applied filters, subject-area constraints, and coverage periods for each of the prominent databases are documented in Table 2 to empower full search reproducibility, predominantly across IEEE Xplore, SpringerLink, Elsevier ScienceDirect, Wiley Online Library, and MDPI.
- Duplicate records were removed before title-abstract screening.
- Full-text assessment and data extraction were performed independently by two reviewers.
- Extracted metadata included publication source, model type, input features, dataset, evaluation parameters, and findings. In order to quantify dataset standardization and benchmarking readiness, specifically for publicly available datasets, we define three observable indicators: (1) public accessibility with explicit reuse terms (DOI/handle/repository + license), (2) schema transparency (machine-readable feature/units + label taxonomy + protocol/sensor modality metadata), and (3) reproducible benchmark protocol (canonical split or clearly specified train/test partitioning, with baselines when available).
- Statistical aggregation was employed to summarize the architectural trends and detection outcomes.
- Quality assessment of primary studies (i.e., as exhibited in Figure 2) was conducted using predefined criteria emphasizing methodological rigor, dataset credibility, and experimental reproducibility. Bias risk was evaluated across four structured dimensions: selection bias, assessed through pre-specified dual-reviewer inclusion and exclusion criteria; performance bias, assessed through verification of experimental reproducibility and dataset credibility; detection bias, assessed through evaluation of metric completeness and reporting transparency; and reporting bias, assessed through consistency between stated study objectives and reported outcomes. Each selected article was evaluated for clarity of objectives, transparency in model design, statistical validity of results, and completeness of performance metrics. Studies demonstrating reproducible experimental setups, peer-reviewed validation, and consistent evaluation protocols across UAV cybersecurity tasks were classified as high-quality evidence for synthesis.

**Table 2:** Search strategy for deep learning-based UAV cybersecurity across databases.

Database	Search period	Core keywords/Boolean string	Filters applied	Inclusion focus	Exclusion criteria
IEEE Xplore	2015–2025	(“UAV” OR “drone”) AND (“cybersecurity” OR “intrusion” OR “spoofing” OR “jamming”) AND (“deep learning” OR “CNN” OR “RNN” OR “GAN” OR “reinforcement learning”)	Journals only, English language	Model architecture evolution, detection accuracy	Short notes
SpringerLink	2015–2025	(“UAV” AND “AI security”) OR (“deep neural network” AND “IoD”)	Computer Science, Engineering subject areas	Edge deployment, federated learning, blockchain integration	Non-peer-reviewed or duplicate
Elsevier (ScienceDirect)	2015–2025	(“autonomous aerial vehicle” AND “threat detection”) OR (“deep reinforcement learning” AND “attack mitigation”)	Article type: Research article	Adversarial training, adaptive models	Review or survey papers
Wiley Online Library	2015–2025	(“AI-based UAV defense”) OR (“privacy preserving UAV communication”)	Subject filter: AI and Robotics	Explainability, regulatory compliance, ethical deployment	Patents, editorial materials
MDPI	2015–2025	(“UAV cybersecurity” AND “deep learning intrusion detection”)	Indexed journals only	Model comparison, performance metrics	Preprints, duplicates

It is worth highlighting that the selected research studies were reviewed in two clear stages by independent reviewers. First, the titles and abstracts were screened, followed by a detailed full-text review. Any disagreements were discussed until a consensus was reached. Hereby, the Figure 2 illustrates the PRISMA protocol with the record counts at each stage. Initially, 849 records were identified from multiple sources, including IEEE Xplore (158), ACM Digital Library (62), SpringerLink (144), ScienceDirect (151), Scopus (188), and other sources (146). After removing 273 duplicate records, 576 unique studies remained for title and abstract screening. This step excluded 421 studies and moved 155 articles forward for full-text assessment. During the full-text review, 98 articles were excluded for specific reasons, such as not using deep learning methods (64), lacking quantitative evaluation (12), being published in non-English language (10), or having unavailable full text (12). Ultimately, 57 studies were retained for qualitative synthesis and formed the basis for further analysis. The extracted information included but not limited to: model types, architectural details, datasets, evaluation metrics such as accuracy, precision, recall, F1-score, AUC, and reported computational cost.

## Analysis based on research questions

### Evolution of deep learning architectures for UAV cybersecurity (RQ-1)

CNN-based intrusion detection models have achieved near-perfect accuracy in identifying malicious UAV activities by leveraging spatial feature extraction (i.e., as illustrated in Table 3). Deep CNN classifiers can distinguish anomalous network traffic or sensor inputs with high precision, as evidenced by a ConvNet achieving 99.5% detection accuracy with only millisecond-level latency (Al-Haija and Badawi, 2022). These accuracy and latency values are reported under each source paper’s dataset and evaluation setting. In context of Table 3, a widely reused open-source benchmark for encrypted UAV Wi-Fi traffic is UAV-IDS-2020, which specifies 55 engineered statistical features (54 + label in bidirectional mode) and 68,736 labelled samples spanning three commercial UAV types (Parrot Bebop, DBPower UDI, DJI Spark) and two flow modes (unidirectional/bidirectional), including feature-generation and feature-dependency metadata (runtime and incidence/graph-style dependencies) that accredit reproducible baselines rather than dataset-opaque accuracy claims. Thus, the cited evidence is based on Wi-Fi traffic intrusion data, so these metrics should be interpreted as within-investigated research results rather than a shared UAV cybersecurity benchmark. We also observed that cross-study comparison remains limited because the reported gains are tied to task-specific data sources, feature schemas, and validation settings. Reported values from Wi-Fi intrusion datasets do not establish direct equivalence with telemetry, RF, or swarm-level studies. Standardized benchmarking remains concentrated in a small subset of public datasets, which narrows fair comparison across the broader literature. Recent research shows that architectural optimizations like knowledge distillation and pruning can drastically reduce model size and inference time without sacrificing accuracy (Arthur, 2019). These CNN-driven approaches thus provide fast and reliable threat recognition for UAV systems, although their deployment on resource-limited drones requires careful model compression, herewith, the Table 4 illustrates that the RNN/LSTM (Long short-term memory) frameworks facilitate sequential analysis of UAV telemetry and communication streams to capture temporal patterns indicative of attacks. Long Short-Term Memory networks have been used to predict expected sensor readings and detect anomalies when actual readings deviate to attain about 99% accuracy in UAV fault/attack detection (Zhou et al., 2025; Yang et al., 2025). Such recurrent models excel at detecting gradual or time-dependent intrusions (e.g., progressive sensor spoofing) by maintaining memory of past states. Hybrid CNN-LSTM schemes further improve performance by combining spatial feature learning with temporal context (Miao et al., 2024), which accelerates detection and focuses on relevant sequence features.

**Table 3:** CNN-based deep learning models for UAV cybersecurity.

Publication	Model type	Threat addressed	Evaluation metrics	Architectural features
Al-Haija and Badawi (2022)	CNN (UAV-IDS-ConvNet)	Network intrusion (Wi-Fi traffic)	99.5% detection accuracy; 2.77 ms inference latency	Deep CNN trained on encrypted traffic; real-time binary classifier for UAV network anomalies
Medhi et al. (2025)	Pruned DNN (UAV-DiPNID)	Network intrusion (Wi-Fi traffic)	99.61% accuracy; 80.70% lower inference time; 90% smaller model	Knowledge distillation + weight pruning for compact CNN-based IDS; incremental learning for evolving attacks (Arthur, 2019)

**Table 4:** RNN/LSTM frameworks for sequential UAV anomaly detection.

Publication	Model type	Threat addressed	Evaluation metrics	Architectural features
Miao et al. (2024)	CNN + Bi-LSTM (Attention)	Network intrusion (UAV communication)	High detection accuracy; reduced execution time vs. pure CNN	CNN feature extractor with Bi-LSTM sequence modeling; attention mechanism focuses on salient traffic features
He et al. (2022)	LSTM + CGAN (FL-based)	Network intrusion (distributed)	Improved detection rate and generalization under data scarcity	LSTM classifier augmented by GAN-synthesized samples; blockchain-federated learning for cross-UAV IDS integration

**Figure 3:**
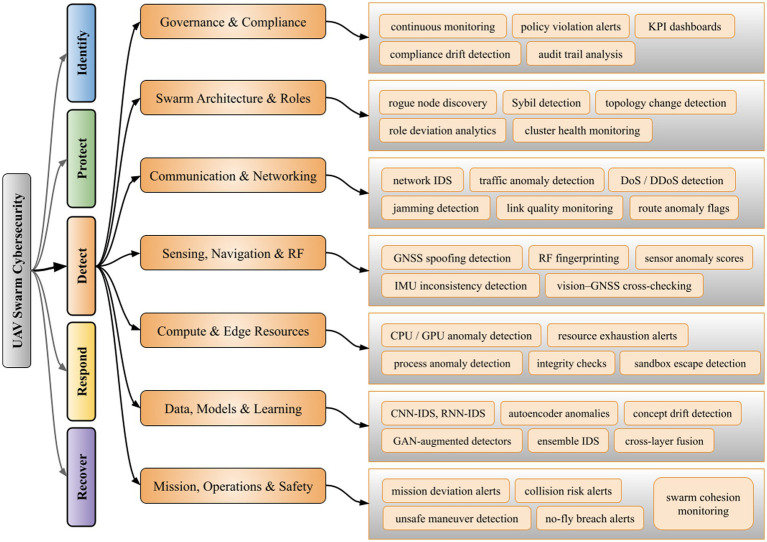
Taxonomy of detect functions for UAV swarm cybersecurity.

During our investigation, we observed that the autoencoder and GAN-based methods address UAV cybersecurity in scenarios with limited or no labeled attack data by operating in unsupervised or adversarial fashions (Table 5). Deep autoencoders trained on normal UAV behavior can flag deviations (e.g., unusual flight kinematics under GNSS spoofing or jamming) via reconstruction error spikes (Da Silva and Branco, 2025). This allows detection of zero-day attacks by learning an implicit model of ‘normal’ and sensing when an input strongly diverges. To enhance such anomaly detectors, optimization algorithms have been used to tune model parameters and select features with an aim to yield higher intrusion detection rates in IoT-assisted UAV networks (Negm et al., 2024). Generative Adversarial Networks have also been leveraged to expand attack training data, for instance, synthesizing realistic malicious communication sequences which significantly improves detection of both known and novel threats (Yoo et al., 2024). These unsupervised and generative approaches increase the robustness of UAV defenses (i.e., as specified in Figure 3), though at the cost of added model complexity and training effort.

**Table 5:** Autoencoder and GAN models for unsupervised UAV threat detection.

Publication	Model type	Threat addressed	Evaluation metrics	Architectural features
Negm et al. (2024)	Deep Autoencoder + Tasmanian Devil Optimization (TDO)	Network intrusion (IoT-UAV network)	Optimized detection accuracy (meta-heuristic tuning)	TDO fine-tunes autoencoder parameters; focuses on UAV network traffic features to improve IDS accuracy
Yoo et al. (2024)	GAN (data augmentation)	UAV cyber-attacks (intrusion dataset)	+37% detection (known attacks); +30% (unknown attacks) via GAN-generated data	Used five GANs: SeqGAN, MaskGAN, RankGAN, StepGAN, and LeakGAN to synthesize realistic UAV communication sequences; augments training data to boost IDS coverage of rare attack patterns

We also noticed and exhibited in Table 6 that deep reinforcement learning offers UAVs an adaptive defense mechanism by actively learning how to respond to attacks through trial-and-error. In an RL-based IDS, the drone or network agent is trained to maximize a reward for correctly identifying or mitigating intrusions, rather than relying on static detection rules. For instance, a Deep deterministic policy gradient (DDPG) agent can learn to discern and react to network attacks in aerial networks, and generate higher detection ratios than traditional classifiers (Tao et al., 2021). Similarly, Deep Reinforcement Learning (DRL) policies empower UAVs to handle jamming attacks by dynamically adjusting their trajectories or transmission parameters; this has been shown to greatly reduce communication bit-error-rate and conserve power under jammer presence (Liu et al., 2022). These results demonstrate that UAVs can autonomously optimize their behavior to maintain security in changing threat environments. The effectiveness of DRL-based defense, however, hinges on well-designed reward functions and efficient onboard computation, as training or running deep agents on UAV hardware remains challenging.

**Table 6:** Deep reinforcement learning for autonomous UAV cyber defense.

Publication	Model type	Threat addressed	Evaluation metrics	Architectural features
Tao et al. (2021)	DDPG (actor-critic RL)	Network intrusion (aerial)	Higher attack detection ratio vs. non-learning IDS	Agent learns intrusion signatures via continuous action policy; reward function tuned for maximizing true detection in UAV network
Liu et al. (2022)	Dueling DQN (Deep Q-Net)	Jamming attack (comms link)	~15–20% lower BER (Bit-Error-Rate); improved energy efficiency vs. baseline	UAV relay adjusts path and power in real-time to evade jamming; dueling neural network estimates Q-values for robust anti-jamming decisions

### Optimization and deployment strategies on resource-constrained UAVs (RQ-2)

Various studies have leveraged model compression techniques to enable onboard deep learning in UAVs with limited computing resources. Abdalla et al. (2025) applied iterative pruning and post-training quantization to a DeepLab v3 + model and achieved up to ~75% smaller models (i.e., model size reduction) with minimal (~1–3%) accuracy loss. Xu et al. (2023) similarly compressed a large YOLOv3 object detector for real-time UAV use, and thus has significantly reduced its storage and processing demands (with negligible accuracy drop). Likewise, recent research (published in 2025) introduced the UAV-DiPNID intrusion detection network optimized via both weight pruning and knowledge distillation with the aim to shrink the DNN size by 90% and cutting inference time by ~80% while preserving high detection accuracy (Medhi et al., 2025). In this review, such speedup and ‘high accuracy’ claims are tied to the original study’s evaluation pipeline and deployment assumption. Table 7 indicates that evidence ranges from simulated UAV networks to edge-device tests, so hardware, traffic workload, and test protocol should be matched before comparing or transferring inference-time results across papers. Real-world deployment realism also differs across these reports. Results from simulated UAV networks, dataset-based testing, offline evaluation, edge simulation, and edge-device testing represent different operational conditions and should be read as setting-specific evidence rather than interchangeable proof of field performance. Reported latency, accuracy, and energy values are most informative when the deployment target and evaluation pipeline are aligned. Herewith, Table 7 compares these model compression and distillation strategies and highlights their impact on UAV onboard performance. The investigated compression methods reveal distinct trade-offs. It was observed that the pruning-centric pipelines are most aligned with latency reduction on embedded platforms, while quantization is primarily used as a footprint-reduction step that can introduce a small accuracy penalty in some vision workloads. Moreover, distillation-based compression is most often used when accuracy retention is prioritized, and pairing distillation with pruning yields the strongest quantified latency gains among the listed IDS studies. We also noticed a trend that the energy savings are the least consistently reported dimension. Advantageously, all the compressed models demonstrated improved runtime efficiency on edge hardware (e.g., NVIDIA Jetson), which confirmed the viability of deploying deep neural networks on resource-constrained drones without significant accuracy compromise.

**Table 7:** Lightweight model compression techniques for onboard UAV intrusion detection.

Publication (year)	Latency reduction	Energy savings	High detection accuracy	Onboard deployment feasibility
Medhi et al. (2025)	✔ (≈80%↓)	✗	✔ (≈99.6%)	✔ *(simulated UAV network)*
Wisanwanichthan and Thammawichai (2025)	✔ (7–11% faster)	✔ *(implied)*	✔ (improved by >6%)	✗ *(edge/IoT focus)*
Umar et al. (2025)	✔ (ultra-fast)	✔	✔ (99.4%)	✔ *(edge device test)*

In tandem with compression, researchers are optimizing where and how inference is performed in UAV systems to balance speed, energy, and accuracy. Luo et al. (2022) developed KeepEdge, an edge-intelligence framework that uses cloud-to-UAV knowledge distillation to deploy a lightweight model onboard for visual positioning in drone delivery. The distilled model runs locally on the UAV with significantly reduced latency to allow real-time operation without offloading. For UAV swarms, energy-aware learning has been incorporated to prolong missions: Kim et al. (2024) showed that a Deep Q-network based control policy can cut multi-drone energy use by 33–74% (rural vs. urban scenarios) while maintaining timely data collection, by optimizing trajectories and transmission schedules. Such approaches underscore the importance of onboard edge inference (avoiding reliance on cloud connectivity) and intelligent resource management in swarms. Practical deployment still depends on the target SWaP profile and compute stack of the UAV. A compressed detector validated on one edge board does not transfer uniformly across mixed hardware profiles, battery limits, or payload limits. Communication conditions also shape feasibility because offloading, gradient exchange, and shared summaries depend on link availability and bandwidth limits that differ between single-UAV trials and swarm operations. We have summarized several deployment-time optimizations in Table 8, which include edge inference schemes and energy-aware frameworks. The reported ‘accuracy/precision retention’ and ‘high intrusion detection rates’ come from task-specific datasets and study-specific evaluation settings. Precision and recall should be interpreted together in these deployment reports because a higher alert hit rate often accompanies stricter or looser operating thresholds. In onboard security use, false alarms increase compute, communication, and response burden, whereas missed attacks weaken mission protection. These trade-offs are most informative when metric definitions, thresholds, hardware targets, and test conditions are aligned across studies. Table 8 labels these settings explicitly (e.g., offline evaluation, dataset-based testing, and edge simulation), so comparability is strongest within the same task and test condition. Notably, these strategies do not sacrifice detection capability, e.g., the distilled positioning model in KeepEdge retained over 93% precision, and the UAV-DiPNID security model maintained high intrusion detection rates, thereby guaranteed that UAVs can respond to threats in real time despite their constrained hardware.

**Table 8:** Knowledge distillation strategies and edge inference enhancements for UAV security AI.

Publication (year)	Massive model compression	Latency optimized inference	Accuracy retention	Edge/onboard tested
Wisanwanichthan and Thammawichai (2025)	✔ (≥92% fewer params)	✔ (faster inference)	✔ (no accuracy drop)	✗ (offline evaluation)
Medhi et al. (2025)	✔ (knowledge distillation + pruning)	✔ (real-time)	✔ 99.61%	✗ (dataset-based)
Ma et al. (2025)	✗ (architecture-level)	✔ (split/early exit)	✔ (meets QoS)	✔ (edge simulation)

The outcomes of our in-depth assessment demonstrate that through careful model optimization and deployment strategies, deep learning models can be adapted for real-time, onboard UAV operations even under stringent resource limits. Techniques such as pruning, quantization, and distillation compress models to fit embedded hardware (Medhi et al., 2025; Abdalla et al., 2025), while edge computing frameworks and energy-aware algorithms ensure that UAVs (and UAV swarms) can perform complex inference tasks with limited battery and connectivity (Luo et al., 2022; Kim et al., 2024).

### Hybrid and decentralized deep learning security frameworks (RQ-3)

**Figure 4:**
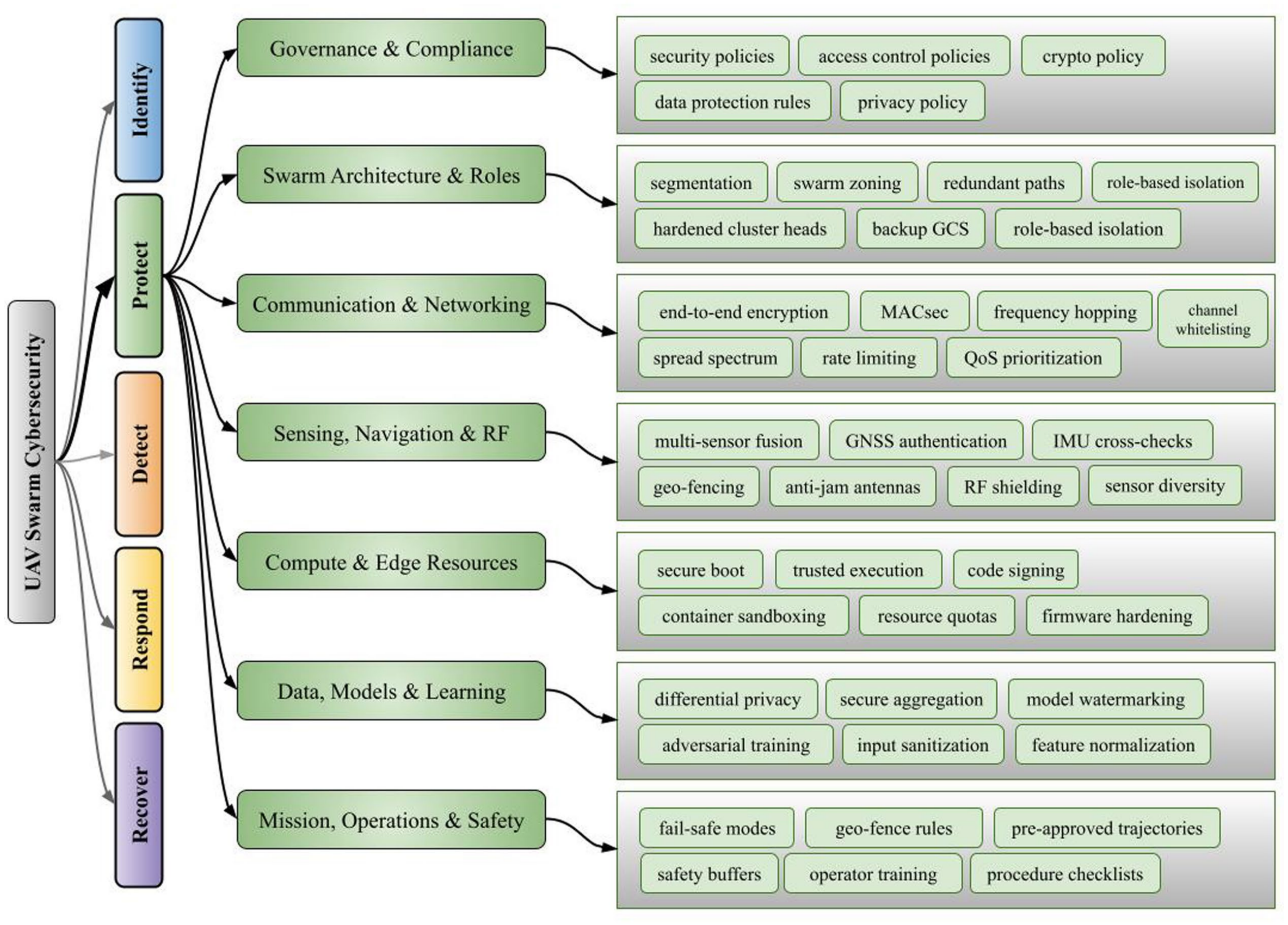
Taxonomy of protect functions for UAV swarm cybersecurity.

Hybrid learning models embedded with decentralized intelligence are progressively deployed to secure UAV networks. These models combine privacy-preserving training with consensus-driven validation to protect UAV systems against distributed and evolving threats (i.e., as identified in Figure 4). For instance:

- Federated and edge-based techniques reduce cloud dependency by assisting in-situ anomaly detection and response.
- Simultaneously, fog-empowered architectures facilitate hierarchical defense pipelines to intercept threats closer to the data’s origin.
- Blockchain further reinforces trust mechanisms across collaborative UAV formations by maintaining immutable logs and validating inter-UAV interactions through smart contracts.

When combined with deep learning, such configurations guarantee authenticated data provenance, continuous integrity validation, and dynamic threat mitigation without centralized control. They also suppress single points of failure, increase system auditability, and enhance multi-agent trust negotiation.

The stated architectural convergence strengthens response latency and secure inter-node communications while allowing scalable threat adaptation. The integration of consensus algorithms, gradient encryption, and fault-tolerant detection filters across these settings collectively improves system reliability in adversarial environments. Recent peer-reviewed studies (Li et al., 2024; Fahim-Ul-Islam et al., 2025; Zhu and Qin, 2024; Alkabir et al., 2025) validate such performance improvements under constrained bandwidth, energy, and adversarial contamination conditions. As shown in Table 9, various architectures implement hybrid models that facilitate distributed learning, secured inference sharing, and cooperative defense orchestration across swarms and edge infrastructures.

**Table 9:** Hybrid-decentralized deep learning-based security models for UAVs.

Citation	Security architecture type	Deployment target	Threat focus	Evaluation results	Technical contribution	Resilience measure
Li et al. (2024)	Blockchain-DL hybrid architecture	UAV swarm network (cluster)	Identity spoofing; unauthorized access; data tampering	Qualitative security analysis (conceptual framework)	Blockchain-based identity management; DL-driven situational awareness; BC-enabled secure data sharing	Tamper-proof data ledger; enhanced cross-UAV trust consensus
Fahim-Ul-Islam et al. (2025)	Resilient FL (gradient clipping + attention-DNN)	UAV network (collaborative IDS)	Model poisoning; backdoor adversarial updates	+7.2% accuracy, +10.1% recall vs. FedAvg/FedProx baselines	Weighted gradient clipping aggregator; attention-enhanced DNN (ANET)	Mitigates malicious model updates; fewer false alarms (improved precision
Zhu and Qin (2024)	Blockchain-integrated FL (UBFL framework)	UAV swarm + edge (MEC servers)	Data poisoning attacks; gradient tampering; privacy leakage	~99.9% detection accuracy and precision; robust under 30% poisoned clients	Nonlinear encrypted gradient sharing; blockchain smart-contract aggregation; RCF anomaly detection filter	Byzantine fault tolerance (withstands 30% malicious nodes); tamper-proof model updates
Alkabir et al. (2025)	Swarm intelligence + edge blockchain	UAV swarm + ground sensors (50-node)	Distributed DoS (flooding) attacks; network intrusions	98.7% DDoS detection accuracy; 47% lower latency vs. FL baseline; 73% less storage use	Ant-colony-based threat dissemination; Hyperledger Fabric ledger for secure logging; IPFS off-chain storage	High throughput ledger (420 blockchain transaction rates per second); 62% fewer false alerts (reduced False Positives rate)

### Adaptive and self-defending deep learning paradigms (RQ-4)

#### Adversarial training and distillation for UAV defense

Deep learning models for UAVs have been fortified using adversarial training (training on perturbed examples) and defensive distillation (using a softened teacher-student training approach). Studies report that these proactive defenses substantially improve resilience of UAV vision and control systems against manipulated inputs (Tian et al., 2021). Reported gains under adversarial pressure remain tied to the attack type, perturbation process, and deployment setting used in each source paper. Results from autopilot simulation and simulated UAV communication networks support resistance under investigated conditions, yet they do not establish equal field performance across sensing modalities or mission profiles. Precision, recall, and error reduction should thus be read with the same attack model and test pipeline in view. For instance, in order to prevent control divergence, the image-based drone navigation networks retrained with adversarial signature dataset and can maintain high accuracy under attack. Likewise, knowledge distillation has produced compact yet robust intrusion detectors that run on-board UAVs with minimal performance loss. Our assessment found that combining adversarially augmented data and distillation-based model hardening results in self-defending UAV AI systems that maintain high detection and control accuracy even against sophisticated input perturbations (Table 10).

**Table 10:** Adversarial training and defensive distillation in UAV cybersecurity.

Citation	Threat type	Model/method	Learning strategy	Evaluation metrics	Deployment target	Contribution
Tian et al. (2021)	Adversarial sensor input attacks (vision-based UAV navigation)	CNN regression (DroneNet)	Adversarial training + defensive distillation	Robust navigation error (reduced deviation under attack) - *robustness improved to near baseline levels*	UAV autopilot simulation	FGSM defense, robustness boost, image spoofing mitigation
Medhi et al. (2025)	Malicious network traffic (UAV intrusion attempts)	Deep CNN + pruning (UAV-DiPNID)	Knowledge distillation (teacher-student)	Accuracy 99.61% (vs. 99.37% baseline); ~80% inference speedup; ~90% model size reduction	UAV communication network (simulated)	distilled IDS, compact DNN, real-time defense

#### Transfer learning and domain adaptation across platforms

Due to variability in operational environments, transfer learning techniques enable UAV cybersecurity models to generalize across domains. Recent research leverages high-fidelity simulations and multi-modal source data to pre-train deep models, which are then fine-tuned on target UAVs with limited real data (Yang et al., 2025). This approach has markedly improved anomaly detection and fault diagnosis in novel UAV platforms to mitigate the data scarcity issue. Similarly, domain adaptation methods align feature distributions across different sensory or network domains. By adversarially reducing domain gaps (e.g., between RF channels or flight regimes), UAV detection models achieve almost invariant performance when deployed in new conditions. These strategies demonstrate that knowledge transfer across simulators, sensor modalities, or UAV types, substantially boosts the robustness and generalization of UAV cyber defense models in practice (Table 11).

**Table 11:** Transfer learning and domain adaptation across UAV platforms.

Citation	Threat type	Model/method	Learning strategy	Evaluation metrics	Deployment target	Contribution
Yang et al. (2025)	UAV flight anomalies	1D CNN-BiLSTM	Multi-source transfer, fine-tuning	~98–99% accuracy, average accuracy: 96%	UAV telemetry (real/sim)	Sim-to-real, fault detection, few-shot tuning
Yang et al. (2025)	Swarm anomaly detection	Meta-GAD	Meta-transfer learning	(high diagnostic accuracy)	UAV swarms (sim/real)	Fast adaptation, low-data regime, meta-finetune

#### Self-supervised and continual learning under dynamic threats

To cope with dynamic, evolving threat conditions, UAV cybersecurity has embraced self-supervised learning and continual learning paradigms. Self-supervised methods learn intrinsic structure from unlabeled UAV data (e.g., nominal flight telemetry) to allow detection of novel anomalies without explicit attack labels. By training on augmented versions of normal data and using proxy tasks (such as predicting transformations), UAV fault detection models can identify deviations that indicate attacks or failures and thus achieve high accuracy even for unforeseen events. In parallel, continual learning frameworks update UAV intrusion detectors incrementally as new attack patterns emerge. Techniques like federated continual learning avoid catastrophic forgetting by preserving past knowledge while integrating new information. This yields adaptive IDS models that sustain high detection rates over time to guarantee UAV networks remain secure against ever-changing threats without frequent full retraining (Table 12).

**Table 12:** Self-supervised and continual learning under dynamic UAV threats.

Citation	Threat type	Model/method	Learning strategy	Deployment target	Contribution
Wang et al. (2024)	UAV faults	Graph VAE + GMAT	Contrastive learning, self-supervision	Fixed-wing UAVs (physical)	augmentation, graph encoder, anomaly score

#### Meta-learning for zero-day threat recognition

**Figure 5:**
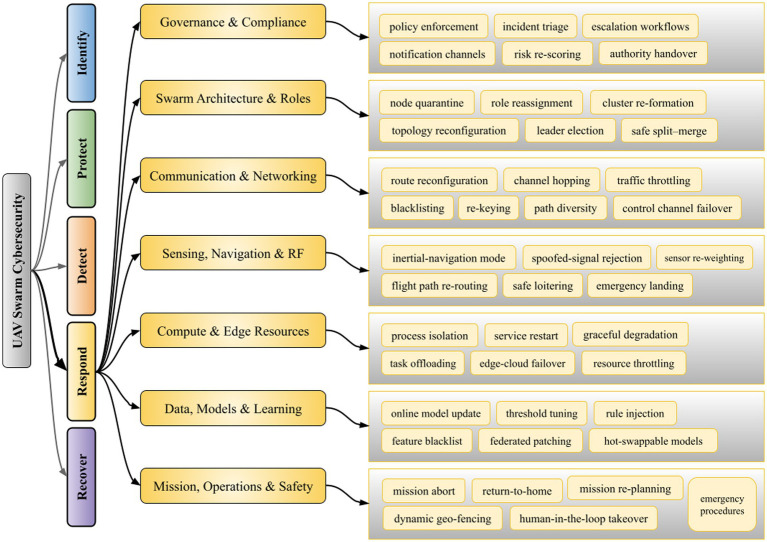
Taxonomy of respond functions for UAV swarm cybersecurity.

Meta-learning approaches have been applied to address zero-day attacks on UAV systems, where labeled signatures of a new threat are scarce or nonexistent. Rather than training a static model, meta-learning ‘learns how to learn’ across many tasks by equipping UAV defense models with rapid adaptation capabilities. A meta-trained anomaly detector can be quickly fine-tuned with only a handful of samples from a new attack, yet achieve high true positive rates in identifying that attack. This few-shot adaptability is crucial for UAV cybersecurity, as zero-day exploits often require immediate recognition before widespread damage (Yan et al., 2024). In the context of Table 2, literature shows that meta-learning-based models are crucial for efficient detection of novel UAV intrusions or faults and require significantly less data and time compared to conventional training. By leveraging prior knowledge from diverse training episodes, meta-learning paradigms furnish UAVs with a form of ‘learning agility’ to recognize and respond to emerging threats in real time (i.e., as recommended in Figure 5).

#### Robustness and generalization benchmarks

We have also observed that to rigorously evaluate UAV deep learning defenses, researchers have begun establishing robustness and generalization benchmarks. These efforts involve testing UAV models across a wide range of adversarial scenarios and operational domains to guarantee consistent performance. For instance, ensemble-based IDS frameworks have demonstrated near-perfect detection across heterogeneous UAV fleets by training on comprehensive, cross-platform datasets (Kabir et al., 2025). These near-perfect results depend on the specific dataset construction and validation protocol used by each benchmark study, including cross-validation as reported in Table 13. They should not be interpreted as a universal performance level across UAV threat types, sensing modalities, or operational domains. Metric comparability also depends on whether studies report threshold-sensitive measures alongside aggregate scores. Accuracy or AUC alone does not resolve the balance between false positives and missed detections across mixed UAV threats. Fair comparison requires matched threat classes, public splits or explicit partitions, and aligned reporting of precision, recall, and attack-conditioned outcomes. Our investigation revealed that the benchmark standardization remained uneven across the reviewed studies. Although, public benchmark use, cross-validation, and explicit protocol reporting improve reproducibility, yet many results still depend on private data, task-specific labels, or mixed simulation and field-like settings. This limits strict cross-paper ranking and supports comparison within matched datasets, threat classes, and evaluation protocols. Also, adversarial robustness benchmarks subject UAV models to worst-case perturbations (e.g., digital and physical attacks) and measure metrics like robust accuracy and attack success rate. It is recommended to follow the best practices (like ensemble learning, cross-validation, and adversarial evaluation) which are needed to deploy reliable, self-defending deep learning paradigms in real-world UAV cybersecurity contexts.

**Table 13:** Robustness and generalization benchmarks for UAV deep learning model.

Citation	Threat type	Model/method	Learning strategy	Evaluation metrics	Deployment target	Contribution
Kabir et al. (2025)	Mixed UAV threats	Gradient Boosting Classifier	Cross-validation	AUC 0.92–1.00	Urban UAVs (heterogeneous)	multi-platform, ensemble, transferability

### Ethical, regulatory, and explainability dimensions (RQ-5)

#### Aviation cybersecurity standards and compliance frameworks

Aviation sector regulations mandate rigorous cybersecurity certification [e.g., RTCA DO-326A/ED-202A and DO-356 define airworthiness security processes and methods (Federal Aviation Administration (FAA)/ European Union Aviation Safety Agency (EASA) (Gorschek and Martins, 2022)]. These standards require multi-layered security analyses and certification for UAV avionics. The FAA’s AI Safety Assurance Roadmap (2024) similarly urges leveraging common technical standards (e.g., NIST CSF, AI RMF) within aviation’s regulatory framework (Boulter, 2024). For learning-enabled UAV IDS functions, this regulatory framing must be implemented as traceable security requirements over the DL dataflow (telemetry/RF features, labels, and federated gradients), because confidentiality or integrity loss in these artifacts directly shifts anomaly scores and can trigger unsafe automated responses. Accordingly, privacy-preserving training and update sharing (e.g., differential privacy, secure multi-party computation, and secure aggregation) becomes a certifiable mitigation that can be claimed in the DO-326A/DO-356 security risk assessment and then verified as ‘objective evidence’ through measurable privacy parameters, cryptographic correctness checks, and attack-informed test cases (e.g., update leakage and tampering attempts). Each model component and dataflow step must map to a documented security requirement. Decision outputs should include associated confidence measures and anomaly logs so that each alert can be verified against regulatory compliance criteria. Compliance scope spans onboard software (DO-178C) through system integrity (DO-326A) to organizational cybersecurity (ISO/IEC 27001, NIST) under civil/military regimes (Kausch et al., 2024). Model architecture decisions from RQ-1 through RQ-4 directly affect the traceability requirements imposed by DO-178C and DO-326A. CNN classifier outputs, federated gradient aggregation schemes, and XAI-generated attribution scores constitute evidence artifacts whose integrity and provenance must be documented within a compliant airworthiness security case. The FAA AI Safety Assurance Roadmap requires each detection decision to be reproducible, bounded in false-alarm rate, and linked to a stated operational assumption, which constrains model design choices including loss function, training data schema, and target hardware. Quantization and pruning steps addressed in RQ-2 carry regulatory significance because any compression step shifting detection thresholds must be verified against the security objectives recorded in the DO-326A risk assessment.

#### Explainable AI (XAI) for UAV cyber defense

We have observed that explainability is increasingly intuit as essential for trustworthy UAV defense. Recent research (Ihekoronye et al., 2024) exhibit that IDS and navigation models should integrate XAI techniques, so operators can audit AI decisions. Herewith, Ihekoronye et al. (2024) recommended using SHAP (SHapley Additive exPlanations)/LIME (Local Interpretable Model-agnostic Explanations) to highlight which telemetry features triggered IDS alerts to make intrusion classification interpretable. Likewise, Minh et al. (2021) referred that embedding XAI in UAV monocular-vision navigation (e.g., obstacle detection models) significantly enhances transparency in flight decisions, by this means, it gains the eligibility to increase trust in AI-driven path planning. Discussed studies advise combining intrinsically transparent models (e.g., decision trees) or post-hoc explainers into UAV security pipelines. Thus, the frameworks presented in (Ihekoronye et al., 2024; Minh et al., 2021) emphasize that leveraging interpretability methods (like SHAP, LIME, or rule extraction) supports the acceptance of automated defenses by stakeholders, and it is applicable at all while preserving technical accuracy.

#### Privacy-preserving and accountable AI mechanisms

Privacy and accountability are addressed by embedding security into the AI pipeline (Table 14). Ntizikira et al. (2023) propose a federated-learning UAV IDS that uses differential privacy and secure multi-party computation to train a global model without exposing local flight data. Their CNN-LSTM network runs locally on each drone and only shares encrypted model updates, guaranteeing data confidentiality. The framework also includes multi-factor authentication (MFA) and automated alert/blacklist mechanisms for accountability. This permits real-time detection while preserving pilot/civilian privacy. These privacy controls can be structured to satisfy airworthiness-security goals by treating ‘flight data confidentiality’ and ‘model-update integrity/provenance’ as explicit security objectives, then mapping them to concrete controls (DP/SMPC/secure aggregation) and to verification outputs that regulators can audit. Operational barriers remain when these controls are added without matching onboard compute and bandwidth limits. Encrypted updates, secure aggregation, and authenticated alert handling increase processing and link overhead, so deployment claims are strongest when the target hardware, channel conditions, and audit path are stated together. Regulatory acceptance also depends on traceable model states and alert records at run time, not only on detection scores reported in isolation. In parallel, accountability mechanisms can be elevated from basic logging to certification-grade traceability by binding each alert to the exact model version, training round, and authenticated actor action (e.g., MFA-gated acknowledgement), with cryptographically chained audit records that support post-incident forensics and security-case maintenance across software baselines.

**Table 14:** Privacy-preserving learning and accountability mechanisms in UAV cybersecurity AI systems.

Citation	Techniques	Protected data	Guarantee mechanism	UAV scenario
Ntizikira et al. (2023)	Federated Learning; Differential Privacy; Secure Multi-party Computation; Multi-Factor Auth.	Onboard sensor logs, telemetry feeds	Model updates aggregated securely; differential privacy (DP) noise added; MFA for IDS database	Smart-city drone networks (collaborative intrusion detection)

#### Societal trust, transparency, and policy implications

Policy-level analyses highlight transparency and trust as critical for UAV AI adoption. Jemielniak (Jemielniak, 2025) argues that mandatory AI transparency (e.g., requiring robots/drones to reveal AI involvement) is needed to preserve public trust. Similarly, the FAA’s AI roadmap explicitly states that AI adoption and acceptance depend on public trust and recommends engagement with policy and standards to guarantee safety (Boulter, 2024). At the city level, privacy-centered designs like the Privadome concept Pillai et al. (2024) propose regulatory-enforced ‘privacy domes’ around citizens to control UAV cameras, and eventually align drone operation with international privacy norms. In stated context, the Table 15 summarizes such perspectives: it lists works focusing on stakeholder trust, algorithmic transparency requirements, and proposed governance measures. Collectively, these sources stress that enforceable explainability (e.g., clear AI disclosure laws) and data protection standards are essential to reconcile UAV AI deployment with societal and legal expectations.

**Table 15:** Governance models and public-trust measures for responsible deployment of AI-enabled UAV systems.

Citation	Focus area	Governance/policy	Societal objective
Jemielniak (2025)	AI transparency	Fourth law of robotics; AI disclosure standards	Enhancing public trust by requiring AI-generated content labeling and system transparency.
Boulter (2024)	Trust in AI	Align with federal AI policy (EO13859)	Emphasizing that public confidence is a prerequisite for safe, wide AI use in aviation.
Pillai et al. (2024)	Privacy/regulation	Privacy-protective drone protocols (Privadome)	Empowering citizens with control over drone data; maintain trust via adherence to international norms.
Heidari et al. (2023)	Trust in secure channel	Zero-knowledge Proof; Transfer Learning; Space Complexity.	Examine the use of blockchain to enable decentralized predictive analytics for efficiently deploying and sharing deep learning methods in a distributed environment.

## Synthesis and comparative discussion

### Cross-RQ analysis and emerging patterns

Findings across RQ-1 to RQ-5 indicate increasing coupling between deep learning model design and UAV system architecture. Detection accuracy, energy efficiency, and governance constraints are treated as joint design axes rather than isolated objectives. This joint view links sensing, communication, and decision layers in a single security stack.

- In this setting, architectural advances in RQ-1 align with RQ-2 results through compressed CNN, RNN, and attention blocks tuned for SWaP-constrained (i.e., Size, Weight, and Power) inference on mixed UAV hardware. Table 16 summarizes this comparison by linking the main model classes to their reported datasets, input features, and practical deployment limits across the reviewed studies. The same comparison also shows that openly specified benchmarking is concentrated in the CNN line built around UAV-IDS-2020, whereas several other model families are summarized through study-specific sources whose public accessibility is not stated in the synthesis tables, including UAV communication data, distributed intrusion data, IoT-UAV intrusion data, simulated UAV networks, mixed real/sim telemetry, and smart-city collaborative IDS settings. Dataset quality is strongest when feature schema, label structure, and protocol metadata are stated explicitly, as in the Wi-Fi benchmark with defined engineered features, labelled samples, UAV platforms, and flow modes. By contrast, access-unspecified and simulation-defined sources provide narrower visibility into data provenance and fixed partitioning, which weakens direct replication across studies. Class imbalance is reflected by the reported use of GAN-generated attack sequences for rare attack patterns and by few-shot adaptation in low-data regimes. Reproducibility remains strongest when public accessibility, schema transparency, and explicit train/test partitioning are reported together, yet these conditions are not described with equal detail across the reviewed evidence. We recommend that these dataset-level differences should be considered before linking model gains to later swarm, edge, and adaptive-defense findings (Table 16).
- Building on this trend, RQ-3 shows these models embedded in federated and edge-centered security stacks, where shared gradients and encrypted summaries preserve swarm-wide situational awareness under bandwidth limits.
- Extending this linkage, RQ-4 highlights self-defending training regimes in which adversarial, self-supervised, and meta-learned encoders reuse the same telemetry and packet features optimized for deployment.
- From the same evidence base, mission-level coordination layers in RQ-3 couple with RQ-4 policies so that DRL agents adapt flight paths and routing strategies while maintaining integrity guarantees from cryptographic trust anchors.
- In parallel, RQ-5 connects these technical layers to certification, explainability, and privacy requirements, which steer model selection toward architectures whose internal scores and alerts remain auditable by regulators and operators. Translating this alignment into practice requires model design choices to be recorded as traceable security claims within DO-326A and DO-356 assurance cases. The compression ratio accepted during pruning, the aggregation rule applied in federated rounds, and the XAI attribution method used at inference all determine whether a deployed IDS produces outputs auditable under airworthiness certification criteria. FAA AI Safety Assurance guidance (Boulter, 2024) specifies that learning-enabled functions must demonstrate bounded decision uncertainty and reproducible test outcomes across the intended operational conditions, which ties the evaluation protocols described in RQ-1 through RQ-4 directly to regulatory acceptance criteria.

**Table 16:** Comparative summary of models, datasets, features, and deployment limits in deep learning-based UAV cybersecurity.

Model class	Representative model(s)	Dataset/data source	Input features	Deployment limits
CNN	UAV-IDS-ConvNet (Al-Haija and Badawi, 2022); UAV-DiPNID (Medhi et al., 2025; Arthur, 2019)	Wi-Fi traffic; UAV-IDS-2020 benchmark	Encrypted UAV Wi-Fi traffic; 55 engineered statistical features	Needs compression for resource-limited UAVs; pruning/distillation used to reduce size and inference time
RNN/LSTM	CNN + Bi-LSTM (Miao et al., 2024); LSTM + CGAN (He et al., 2022)	UAV communication/distributed intrusion data	Sequential telemetry and communication streams	Real-time use depends on efficient sequence processing on constrained hardware
Autoencoder/GAN	Deep Autoencoder + TDO (Negm et al., 2024); GAN augmentation (Yoo et al., 2024)	IoT-UAV intrusion data/UAV intrusion dataset	UAV network traffic features; generated attack sequences	Higher training complexity and added model/training effort
DRL	DDPG (Tao et al., 2021); Dueling DQN (Liu et al., 2022)	Aerial intrusion setting/jamming scenario	State-action, communication, path/power adaptation signals	Depends on reward design and efficient onboard computation
Compressed edge models	UAV-DiPNID (Medhi et al., 2025; Arthur, 2019); pruning/quantization/distillation studies [35]–(Ma et al., 2025)	Simulated UAV network; dataset-based testing; edge simulation	Task-specific traffic or perception features	SWaP, latency, energy, and hardware variability constrain transferability
Hybrid decentralized models	Resilient FL (Fahim-Ul-Islam et al., 2025); UBFL (Zhu and Qin, 2024); blockchain-DL (Li et al., 2024; Alkabir et al., 2025)	Collaborative UAV IDS/swarm-edge settings	Shared gradients, encrypted updates, learned anomaly features	Bandwidth, energy, poisoning resilience, and blockchain overhead
Privacy-preserving models	FL + DP + SMPC + MFA (Ntizikira et al., 2023)	Smart-city collaborative drone IDS	Onboard sensor logs, telemetry feeds	Added privacy and verification overhead

### Identified gaps and open challenges

Across RQ-1 to RQ-5, several structural weaknesses remain visible in current DL-based UAV security research. Many designs/models/methods assume ideal telemetry, trusted training data, and uniform hardware profiles that rarely match fleet deployments. These assumptions limit transfer from prototypes to certified aerial platforms. The same transfer gap appears when latency, energy, and accuracy are reported without a full description of onboard processors, battery limits, and communication settings. Evidence from single-UAV testbeds with simplified channel and interference models also leaves swarm-scale deployment under link disruption insufficiently resolved. This practical shortfall links directly to certification because certifiable alerts require auditable outputs under the operating conditions expected in flight. Thus, it is evident that:

- In framework context, RQ-1 architectures seldom expose common intermediate representations across sensing and networking layers, which restricts reuse for optimization tasks addressed in RQ-2.
- Building on that limitation, deployment studies in RQ-2 often rely on single-UAV testbeds with simplified channel and interference models, which gives limited evidence for RQ-3 requirements on swarm-scale trust management.
- Extending the gap further, adaptive methods in RQ-4 rarely include full adversarial pipelines against federated or blockchain-backed training schemes, and it usually leaves poisoning, backdoor, and collusion behaviors insufficiently characterized.
- From a governance angle, links between RQ-5 standards and concrete loss functions, training constraints, or XAI interfaces stay weak, which slows progress toward certifiable anomaly scores and alerts.
- Correspondingly, shared datasets almost never encode privacy resources, audit logs, or operator feedback, so system-level evaluations overlook life-cycle accountability and operator oversight despite strong emphasis in RQ-5.

### Future research directions

We conceptualize that future work needs tight coupling between deep learning innovation, system integration, and regulatory assurance across all identified and elaborated research questions (i.e., RQ-1 to RQ-5). Security solutions must handle heterogeneous fleets, imperfect telemetry, and adversarial behavior while still fitting strict flight, safety, and privacy requirements. This calls for coordinated agendas that join architecture design with certification-ready evaluation.

- In this direction, joint RQ-1 and RQ-2 research should design multi-task detectors that share compressed backbones across RF, GNSS, IMU, and vision streams while meeting latency and energy envelopes on diverse UAV compute stacks.
- Building on that foundation, RQ-3 agendas need swarm-oriented learning architectures that combine federated optimization, coded consensus, and blockchain logging so that inter-UAV trust scores and model states remain globally consistent under link disruption.
- Extending the same goals, RQ-4 work should integrate adversarial training, meta-learning, and continual learning inside hardware-in-the-loop digital twins, tuning policies for spoofing, jamming, and malware scenarios under realistic packet loss and sensor noise.
- From the regulatory side, future RQ-5 studies ought to codify links between aviation standards, XAI explanations, and formal guarantees on detector false-alarm and miss rates to empower machine-readable assurance cases for deep learning-based intrusion and spoofing defenses.

**Figure 6:**
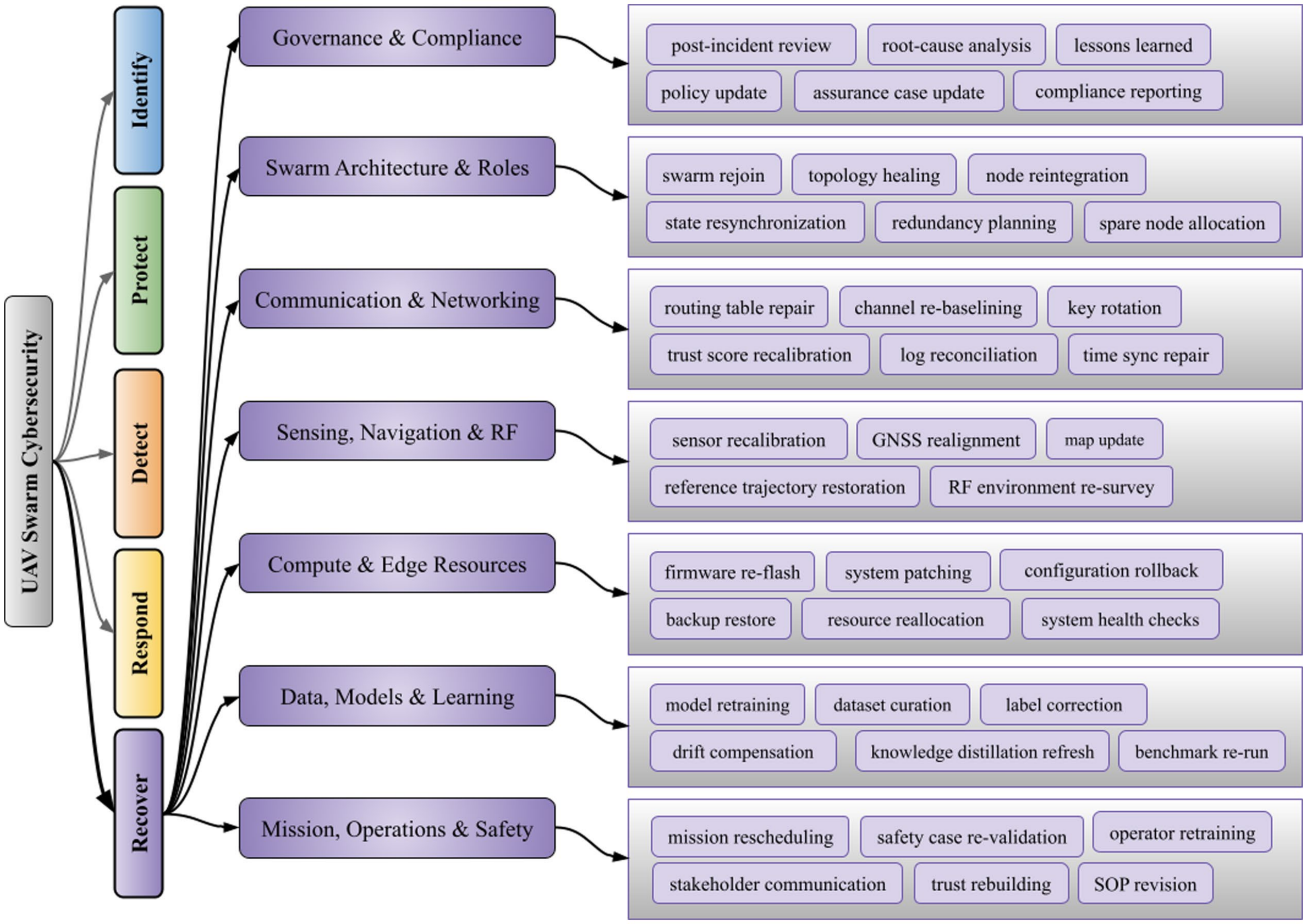
Taxonomy of recover functions for UAV swarm cybersecurity.

We reckon that in order to make the indicated research directions operational, a restructured recovery plan is needed, so swarm operations return to a stable state once detection, protection, and response tasks finish. Herewith, the Figure 6 groups recover functions for UAV swarms into swarm state repair, cryptographic and routing renewal, mission reshaping, and forensic feedback across vehicles, coordinators, and supporting infrastructure, so RQ-1 to RQ-5 conclude with concrete procedures for restoring secure flight.

## Conclusion

Projected systematic review examines predominately past 10 years of deep learning research for unmanned aerial vehicle cybersecurity across intrusion, spoofing, jamming, malware, and coordinated swarm attacks. Within this review, RQ-1 organizes model design into CNN and RNN classifiers, generative and self-supervised models, and deep reinforcement agents for autonomous response. From this basis, RQ-2 addresses compression, quantization, distillation, neural accelerators and split inference for strict SWaP limits and real-time intrusion detection on-board and at the edge. Building on these deployment advances, RQ-3 concentrates on hybrid centralized and decentralized security schemes based on federated learning, fog computing, and blockchain anchored trust for cross-UAV decision sharing and tamper resistance. Extending the same line of reasoning, RQ-4 investigates adaptive and self-defending training pipelines, with adversarial examples, continual and meta-learning facilitating rapid generalization across missions, platforms, and environments. In parallel, RQ-5 links technical progress with aviation safety regulation, explainable security decisions, privacy preservation, and accountability of AI-enabled defense. We paid especial focus that if taken together RQ-1 to RQ-5, the review must show convergence between model design, system-level architecture, swarm coordination, and mission assurance requirements. It also reveals persistent exposure to adaptive adversaries, poisoned updates, cross-layer spoofing, and sensor fusion failures under constrained data sharing and partial observability. Thus, in stated context, the future research needs integrated benchmarks, shared telemetry corpora, certifiable training and verification pipelines, and co-design with regulators and air traffic stakeholders to align deep learning security objectives with certification, safety, and public trust constraints.

## Data Availability

The original contributions presented in the study are included in the article/supplementary material, further inquiries can be directed to the corresponding author.
